# Use, perception, and local management of *Copernicia prunifera* (Miller) H. E. Moore in rural communities in the Brazilian Savanna

**DOI:** 10.1186/s13002-021-00440-5

**Published:** 2021-03-22

**Authors:** José Afonso Santana de Almeilda, Nágila Alves Feitosa, Leilane de Carvalho e Sousa, Raimundo Nonato Oliveira Silva, Rodrigo Ferreira de Morais, Júlio Marcelino Monteiro, José Ribamar de Sousa Júnior

**Affiliations:** 1 Ethnobiology and Conservation Laboratory (LECON), Federal University of Piauí/Campus Amílcar Ferreira Sobral (UFPI/CAFS), BR 343, km 3.5 s/n Meladão, Floriano, Piauí 64808-605 Brazil; 2 Botany Laboratory, State University of Piauí (UESPI)/Coordination of Biological Sciences, Campus of Corrente, Av. Dom Pedro II, 629, Corrente, Piauí 64980-000 Brazil

**Keywords:** Ethnobotany, Carnaúba, Brazilian Savanna, Caatinga, Palm wax

## Abstract

**Background:**

*Copernicia prunifera* belongs to the Arecaceae family, and its production chain includes a set of economic activities based on the use of the stipe, petiole, fiber, fruits, roots, and leaves from which carnaúba wax is extracted, an economically valuable resource in the region. This study aimed to evaluate the uses, management, and perception of the species by local extractors.

**Methods:**

Two communities were studied, *Bem Quer*, where 15 extractors of carnaúba leaves were interviewed, and *Cana*, where 21 extractors considered specialists were interviewed, totaling a sample of 36 interviewees. Interviewees were asked questions about uses, ways of handling, and perception of morphological variation in the carnaúba leaves. The number of leaves extracted and the income obtained from the sale of leaves were estimated from interviews and notes that each leader of extractors held during the year of the research and previous years, as well as direct observations made by researchers in the communities which recollection area of straw hold about 80 thousand individuals of *C. prunifera*. A regression analysis was used to explore the relationships between social variables (age, time in extractive activity, and income obtained from extraction) with the number of leaves exploited.

**Results:**

The leaf was indicated as the most used part, from which an important powder is extracted for the production of wax. In addition, the leaf was also indicated to be used for fertilization and construction. The relationship between the socioeconomic variables, income from extraction, and the number of leaves extracted (in thousands) was significant (*R*^2^ = 0.73 and *p* < 0.001). However, the other variables analyzed in this study, such as the time spent extracting leaves and the years of residence in the community (*R*^2^ = 0.03 and *p* > 0.05); the number of leaves extracted and interviewee age (*R*^2^= 0.05 and *p* > 0.05); and the number of leaves extracted and extraction time (*R*^2^ = 0.04 and *p* > 0.05) did not indicate a relationship.

**Conclusion:**

Local extractors observed that new leaves have the highest sales value, as they have the highest production of powder. In addition, economic factor is the preponderant force that directs the management strategies of native species. For this species, however, morphological and genetic studies are needed for further clarification.

## Background

The management of tree species has been a very common practice in local communities, and studies are necessary in order to understand the role of this human action on plant populations. In this context, ethnobotany is a science that has contributed to understanding the interrelationships between humans and plants, which allows us to understand their benefits [1, 2], in addition to the inherent processes of this interaction. Thus, several ethnobotanical studies have addressed the management of plant species, especially trees, in order to understand the interaction between people and plants [3, 4] from a local management perspective, which can be defined as practices which aim to transform and/or adapt ecosystems, their natural components, and the processes involved in this relationship [5].

The term “management” is related to all human activities transforming and/or maintaining nature in a given state according to plan or purpose [6]. Thus, according to González-Insuasti and Caballero [7], plant management can also be defined as actions or practices carried out by people directly or indirectly on populations or individuals within communities, aiming to favor the availability of a specific characteristic [6]. Additionally, two plant aspects are taken into account for this definition: the selective (when individual plants are favored through human manipulation) and the non-selective (when plant individuals are opportunistically managed); moreover, these practices are carried out in several different environments, from the natural to anthropogenic regions [7]. Another important concept that is often related to traditional management is that of artificial selection, which consists of selective practices that aim to increase and guarantee the availability of individuals with desirable characteristics, and sometimes, there may even be the elimination of undesirable phenotypes [7].

In recent years, some studies have addressed the management of tree species, as well as management of cacti in Mesoamerica, which identified levels of management of plant populations by local communities: collection, *incipient* management, and cultivation [6–8]. Within incipient management, the following types stand out: tolerance (maintenance of individuals in areas destined for agriculture/livestock), protection (elimination of competitors and/or predators), promotion (increase in population density), and ex situ cultivation (propagation of plants in anthropogenic areas) [7].

Likewise, among the main examples of tree species studied in Brazil is the umbuzeiro (*Spondias tuberosa* Arruda), whose fruit is the most used part and presents a desirable selection tendency in relation to size [6, 9, 10]. Another Brazilian example is the fruit of the pineapple guava (*Acca selowiana* [Berg] Burret), whose weight is a characteristic with selection tendency (that is, the target of selection and management) among others [7, 9]. Furthermore, the Brazilian pine (*Araucaria angustifolia* [Bertol.] Kuntze) also presents a larger fruit size as a selection tendency [6, 11]. Another Brazilian species worth mentioning is the Pequi (*Caryocar coriaceum* Wittm), which has a variation in fruit morphology, where size is indicated as the most desired characteristic [12]. Moreover, the fruit is characterized as being fleshy (yellow-colored pulp) [12, 13].

Within this approach, the Arecaceae family has gained prominence regarding the choice of target structures for use and management by human groups, especially the fruit that are targets for management and artificial selection (in other words, human selection). Açaí (*Euterpe oleraceae* Mart.), for example, has peculiar characteristics, such as fruit color and purplish coloration, which are traits resulting from changes [6, 14]. Moreover, the selective characteristic for pupunha (*Bactris gasipaes* Kunth) is size and increase of fruit production, a trait that has changed through artificial selection [6, 15–17]. Despite the various records for the fruit as the morphological structure targeted for selection, many other plant parts are often relevant. For example, unlike the species mentioned above, Butiá (*Butia capitata* [Mart.] Becc) combines all the characteristics of selection in a single resource, the leaf [18]. Although the study did not focus on management, a study on Ouricuri (*Syagrus coronata* [Mart.] Becc) indicated its leaves as an important resource used by the indigenous Fulni-ô people in northeastern Brazil [19].

In addition to the aspects described above, other factors commonly addressed in ethnobotanical studies are socioeconomic factors, such as education, age, gender, occupation, and income [19–21]. Studies on medicinal plants, for example, have indicated that age is an important factor related to knowledge about plants, with older people having more knowledge about medicinal plants compared to younger people [22–24]. For palm trees, Virapongse et al. [25] indicated that the extractors of *Mauritia flexuosa* L.f. leaves are predominantly young, which is related to the fact that younger extractors are better able to climb the palms to remove the leaves. In addition, Moraes et al. [26] demonstrated the great economic importance of *M. flexuosa* whose uses are diverse, the main target of extraction being both the fruits (used in human and animal food) and the leaf (used mainly as handicrafts). Campos et al. [19] also found similar results, demonstrating that age differences may influence the level of involvement in collecting Ouricuri leaves (*Syagrus coronata* [Mart.] Becc.). Thus, understanding whether socioeconomic factors affect the management of locally important species is important for better understanding the relationship between people and plants.

In this framework, the carnaubeira (*C. prunifera* [Miller] HE Moore), an endemic palm of Brazil, which is distributed in the semi-arid northeast and north, belonging to the Arecaceae Family, has been used historically by people from the northeast region of Brazil as a source of income due to its economic and cultural importance. The extraction of the ceriferous powder from its leaves is an extractive activity responsible for sustaining several families during the drought period, which extends from July to December [27–29]. It is a highly versatile plant, its fruits serve as food for animals, its stem (stipe) can be used in the construction of houses due to its resistance and durability, and its fasciculated roots have medicinal properties [30–32]; the leaves are used to make artifacts, forage, and cover shelters for people and animals [32], and one of the most important is its ceriferous powder (carnaúba wax) which is extracted from the leaves. These characteristics have led local people to intensify the leaf extraction from this palm, through actions that aim to enhance the use of the leaves for increased production of wax powder.

Thus, the present work aimed to conduct an ethnobotanical study on carnaúba, in order to characterize the uses and management conducted by local extractors, as well as to highlight the strategies of collection and local management. This study was designed to answer the following questions: Do local extractors perceive morphological variations in carnaúba that can determine management strategies? Our hypothesis was that people would perceive morphological diversity (size and color of leaves) in carnaúba as related to the probability of obtaining higher production of wax powder. Do social variables favor the management of carnaúba in the studied community? Since the leaves are the target morphological structures for use and selection, we expected to find a correlation between socioeconomic variables and the number of leaves extracted. Lastly, which aspects identified in leaf management are most important for the amount collected?

## Methods

### Study area

This study was conducted in two rural communities from the municipality of Barão de Grajaú, MA, in the Bem Quer community and the Cana community. Both communities are located in the eastern region of Maranhão and the Chapada do Alto Itapecuru microregion, neighboring municipalities of São João dos Patos, Sucupira do Riachão, São Francisco do Maranhão (located in the state of Maranhão), Amarante, Jerumenha, and Floriano (located in the state of Piauí) [33]. Additionally, the predominant vegetation type in the region is a typical transition between cerrado (savannah forest) and caatinga, also containing riparian vegetation along the Parnaíba riverbed close to the communities, and carnaúba forests (also known as carnaubais).

**Fig. 1:**
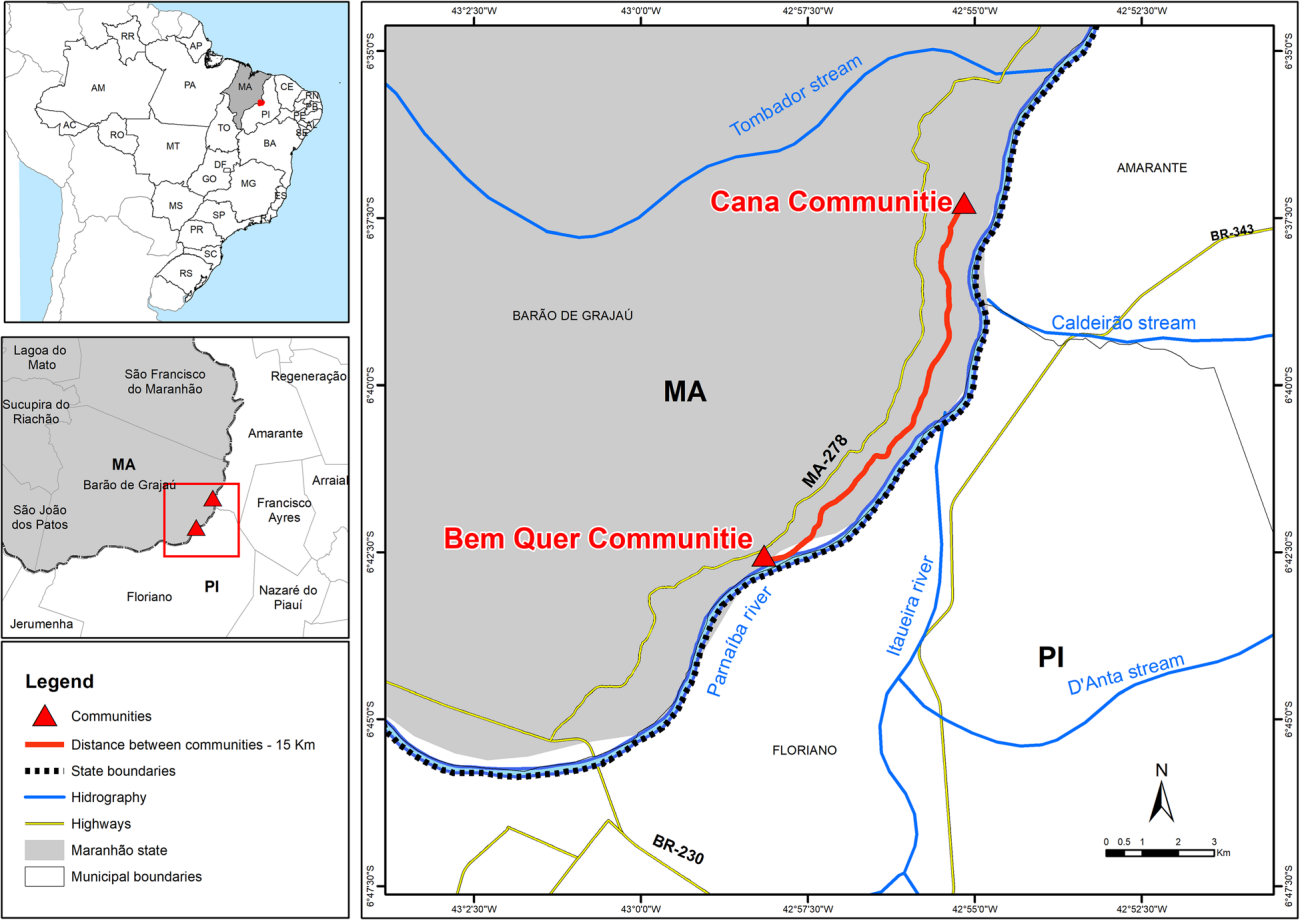
Location of study area, municipality of Barão de Grajaú, Maranhão, Brazil

With an altitude of 108 m and caatinga and cerrado vegetation (Brazilian Savanna), the Bem Quer community has about 56 families according to the information from local health agents. The Cana community has a smaller number of people, about 30 families and 99 inhabitants, according to the data from local health agents. The Cana community is 15 km away from the first community surrounded by similar vegetation types with the main group of active carnaúba extractors active (Fig. 1). In both communities, the main economic activity is agriculture, especially rice, manioc, corn, beans, squash, and watermelon, in addition to fishing activity by some extractors. In addition, the plant populations constitute 30 thousand and 50 thousand individuals in Bem Quer and Cana, respectively. The occurrence area of the carnaúba populations (around 20 km) covers the extension of the Parnaíba river in both communities, constituting vegetal populations with approximately 30 thousand individuals of *C. prunifera* in Bem Quer and 50 thousand in the Cana community.

### Data collection

Traditional knowledge is considered a national heritage in Brazil (Law 13.123/2015). In order to access local knowledge about *C. prunifera*, collaborators (people from the communities) were asked to give their consent by signing the free informed consent form in accordance with resolution No. 292 of 08/07/1999. Thus, this research project was submitted and approved under CAAE registration number: 93417517.3.0000.5660, by the Research Ethics Committee of the Federal University of Piauí (UFPI-CAFS), following the ethical and legal aspects based on resolutions 466/12 and 510/2016 of the National Health Council (CNS).

In order to characterize the knowledge, use, and local management of *C. prunifera*, we used ethnobiological investigation methods based on quantitative and qualitative approaches. Thus, before beginning data collection, we held meetings with members of the target community, in order to clarify the intentions of the study to be developed.

We performed snowball sampling, an intentional non-probabilistic sampling, considering that the people interviewed from the communities were those who maintained the greatest contact (specialists) with carnaúba (*C. prunifera*), that is, through meetings in the community, some extractors are recognized as experts in the process of ripping out “palhas” (leaves), since they deal directly with the species through the annual extraction of the leaves. This sampling method was applied at the end of each interview, where we asked if the interviewee knew another person in the community who has a more comprehensive knowledge of *C. prunifera* [34]. The interviews were based on semi-structured scripts [35], which contained the following questions: (1) What are the uses of the carnaúba palm? (2) Which parts are used? (3) What are the straws (leaves) used for? (4) Are there different types of straw? (5) How many straws (leaves) are needed to obtain one kilogram of wax powder? What is the powder used for? (6) How much straw is collected and how much is sold? (7) How much powder is extracted and how much is sold? (8) How much was your income during the last harvest (or the last time that you extracted powder)? These questions were asked to interviewees from both communities, in addition to other questions about socioeconomic data. Considering the specialist characteristic of the Cana community, where the extractors organize themselves in groups, other questions were only deepened with them, such as (9) Do you notice a morphological variation in the leaves of the carnaúba? (10) During the collection, do you perform leaf selection? (11) Do you perform any type of management (a—simple collection; b—tolerance of arboreal individuals; c—pruning; d—protection against other organisms)? (12) How long have you been engaged in extractive activities?

This work was conducted during two distinct moments. The first moment was during the 2018 harvest season (July to November), when the residents of the Bem Quer community were interviewed and the activity seemed to be in sharp decline according to interviewees. During the second instance, throughout the 2019 harvest (July to November), we were informed about the experts in the Cana community from the interviews conducted in the Bem Quer community, so we also interviewed the specialist extractors who organize themselves into groups in the Cana community. Evidently, the activity of extracting the carnaúba leaves in the region is predominantly performed by men. It is important to note that the Bem Quer community (the one we first contacted) was selected for this study for two main reasons: first, logistically, it is relatively close to UFPI, which favored the development of research by students, considering the scarcity of resources for research; second, other research by our group was already being developed in the community. The Cana community, in turn, was indicated by the extractors of the Bem Quer community, who reported that in the Cana community, there were groups of extractors who were considered specialists.

In addition, we noticed a difference between the two communities regarding leaf extraction. The Bem Quer community presented interviewees who carried out the collection individually (including some who no longer conduct the activity), while in the Cana community, the extractors are organized into groups, with a group coordinator for activities carried out throughout the harvest. The groups in this community are divided into two: one with 10 and the other with 11 local extractors. Thus, 36 interviews were carried out in total, 15 with *C. prunifera* extractors in the Bem Quer community (where the average age was 42.3) and 21 interviews with extractors in the Cana community (where the average age of specialists was 33.8). The number of sampled leaves was estimated from the collection records of the extractors themselves. Due to logistical reasons, we were not able to obtain leaves morphometrical measurements in order to compare (i) variations between each of the two plant populations and (ii) perceptions of the leaf extractors in relation to the measured variations obtained from the previous analysis.

### Data analysis

The data were tabulated in Microsoft Excel. Data analysis was performed from simple proportions using Microsoft Excel. Additionally, for the specialist community, a regression analysis was used to explore the relationships between social variables (age, time in extractive activity, and income obtained from extraction) with the number of leaves exploited. The relationship between time of extraction and time of residence in the community was also analyzed. For these analyses, a *p* < 0.05 was adopted and conducted in the software R 3.6.1 (R Development Core Team 2011).

## Results

### Socioeconomic data, knowledge, and use of *C. prunifera*

**Fig. 2:**
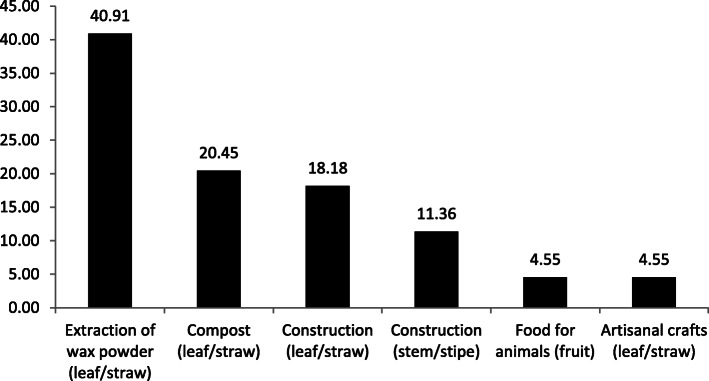
Percentages by use category indicated for *Copernicia prunifera*, Maranhão, Brazil

All interviews indicated the use of carnaúba (*C. prunifera*), the leaf (straw) being the plant structure with more use indications and management, that is, the main part of the plant, followed by the stem (stipe) and then the fruit. The use of the leaf was the most important and expressive (84.09% of the citations), with the main uses cited as fertilization, artisanal crafts, construction (mainly covering houses), and extraction of wax powder (Fig. 2). The straw powder is related to its economic importance, since it produces a type of wax, used to manufacture various objects such as candles, chips, medicine capsules, among other diverse applications, according to the interviews. Besides the use of the leaf (straw), the stem (stipe) of the carnaúba was the second part of the plant (11.36%) with more indications of uses, mainly for the construction of houses (mud houses), fences, and corrals. However, according to the interviewees, this use is in decline. The fruit, in turn, serves as food for animals (4.55%).

**Fig. 3:**
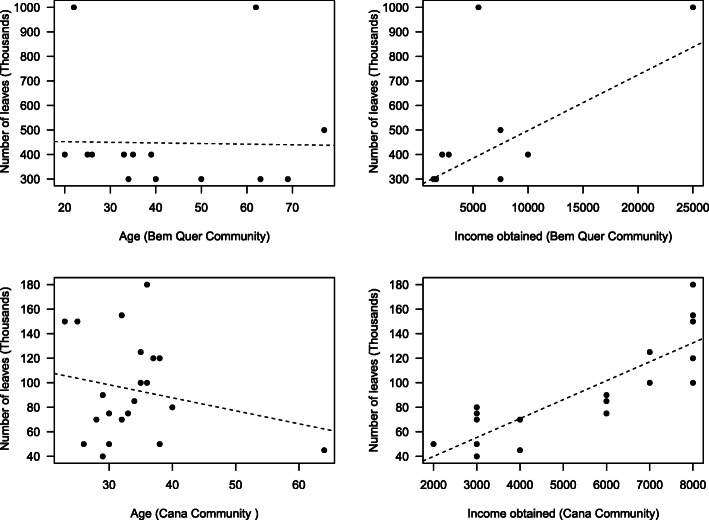
Relationship between the number of leaves extracted and socioeconomic data (age, time in extractive activity, and income obtained from extraction)

As for socioeconomic data, the interviews indicated that there is no relationship between the time the interviewee extracts the leaves and the time of residence in the community (*R*^2^ = 0.03 and *p* > 0.05), between the number of leaves extracted and the age of the interviewees (*R*^2^ = 0.05 and *p* > 0.05), and between the quantity of leaves extracted and extraction time (*R*^2^ = 0.04 and *p* > 0.05). On the other hand, the relationship between income obtained through extraction and the amount of leaves extracted (in thousands) was significant (*R*^2^ = 0.73 and *p* < 0.001) (Fig. 3).

Although the majority of residents from both communities are farmers, it is important to note that carnaúba is an important source of resources, since economically speaking, leaf extraction can generate extra income of approximately eight minimum wages (considering the value of monthly minimum wage for the year 2020, which is R$ 1045.00 or around US$ 246.40 (dollar quote for November 2019), depending on the collection effort of each extractor or group of extractors. Thus, the commercialized product that contributes to the local economy is the ceriferous powder, which is destined through the extractive chain to the industry that produces carnaúba wax from the powder. According to interviewees despite the apparent decline in the uses of other morphological structures of *C. prunifera*, the same occurs in both communities (according to Fig. 2), only the ceriferous powder is locally commercialized. This economic importance is reflected in the organization of extractors, as well as the time dedicated to the extraction, which can reach up to 10 h of daily work.

### Local management and morphological perception of *C. prunifera*

All interviewees claimed to perceive some morphological variation in the leaves of the carnaúba, among which the straw color stood out, with the green color being the most mentioned (42.5% of the total citations), followed by the “dry” color (30%), and lastly, yellow (27.5%). Apparently, the “dry” color, which the interviewees mentioned, is a physiological state of the leaf or a consequence of the action of microorganisms. According to the interviews, it was possible to note that leaf coloring is important for extractors, because, according to them, it influences the production of powder, with green leaves being the most favorable since they produce the most powder.

As for the management of *C. prunifera*, all interviewees performed selection at the time of extraction, with the separation of the “eye” (new leaves that are perceived locally as the structure that produces better quality wax powder, according to the interviewees) from the carnaúba being the main selective action performed by them. In addition, 100% of respondents reported that, when collecting, they eliminate dry straws because they do not produce powder, which is the main extraction product. In addition to the collection, tolerance of plants (maintenance of individuals in agricultural plantation areas, backyards, and farms due to their economic importance) was indicated by all interviewees as a common practice, that is, they stated that they did not cut down the carnaúba, except when the arboreal individuals die, and in this case, the wood is used for construction. Moreover, in relation to the types of management, fire protection was indicated by 85.7% of the interviewees as a deliberate action to prevent burning from causing damage to carnaúba trees (carnaúba forests). However, according to the interviews, the extractors indicated greater concern regarding fires during the collection period, since, in this instance, the collected straws are arranged on the floor for drying and subsequent powder removal, and, in this sense, the fire in the region would destroy all the collection work carried out.

Furthermore, according to the interviewees, the extractors indicated they perform selection according to the following criteria: color, shape, and size of the leaves (straw). Of the extractors, 38.5% indicated they perform leaf selection based on size, due to a relationship, according to them, between size and powder production (“the bigger the straw, the greater the powder production”); 30.8% indicated the shape of the leaf as a selection criterion during collection, and according to them, the “opening” of the straw (fan-shaped leaves) favors the best powder extraction; 26.9% indicated the color as a selection criterion for collection, and according to the interviews, the local specialists said green was the most important criteria. Only one specialist reported not following any selection criteria when carrying out the collection, and he indicated that the most important thing is the production (“the more you collect, the better”). No interviewee indicated the characteristic “odor” or “texture” as selection criteria, which are commonly indicated when the extraction target is fruit. This indicates that the selection criteria are related to the target structures of management use.

**Fig. 4:**
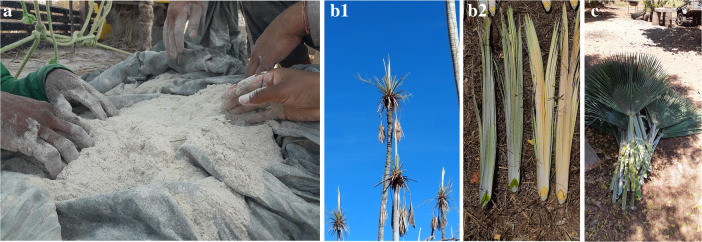
**a** Wax powder. **b1** “Types” of carnaúba *eyes*. **b2** New leaves (immature). **c** “Palhas” (leaves)

When asked if there were individuals of *C. prunifera* from whom nothing was removed, all respondents indicated that only the “eye” of carnaúba was the structure that they sometimes failed to remove. According to the interviews, extractors perceive three types of “eyes” (which correspond to new leaves) from the carnaúba tree as morphological structures that determine their extraction. From the three types (Fig. 4), the two largest “eyes” (larger new leaves) are removed due to the quality of the powder they produce, while the third eye (smaller new leaf) is kept in the carnaúba tree because, according to interviewees, it is from that “eye” all the leaves will regenerate in time for the next collection, which takes place within 1 year.

The powder extracted from the new leaf, or as it is locally called, carnaúba “eye” (leaves still closed and new in the canopy of the carnaúba), has a different color from the powder of the other leaves (open leaves that form the canopy of the carnaúba). A strong implication of this is the difference in the selling price of each type. Although its production is lower, the powder from the carnaúba “eye” has a higher value than the powder from the straw leaves. In addition, our interviews indicated another mechanism that can also be evaluated as another management strategy: not removing any leaves from some individuals of *C. prunifera* during 1 year so that they have more leaf straws with powder the following year. This mechanism is related to the extraction product of the leaves, which is the wax powder. The differences in prices observed from the interviews indicate that the handling of the leaves also aims at a better quality powder. According to the interviews, the powder from the “eye” of the carnaúba straw (closed leaves and new ones existing in the canopy of the carnaúba) has higher quality and therefore higher market value than the powder from the rest of the straws (open leaves that form the carnaúba canopy).

According to the interviews, the number of straw leaves collected per year varies between 150 and 600 thousand leaves in the Bem Quer community and from 200 to 900 thousand in the Cana community (Table 1), depending on the collection effort of the extractor (or group of extractors), and the previous year’s collection activity. The same goes for the amount of straw collected per day, which ranged on average between 20 to 30 thousand in the Bem Quer community and 20 to 50 thousand in the Cana community. In order to extract 1 kg of powder from the carnaúba straw, 1000 straws are needed in both communities, according to the interviews. The value of the powder varied between R$ 2.50 and 6.00 in the Bem Quer community and between R$ 7.00 and 8.00 in the Cana community, and all products derived from it are produced by local manufacturers, which means the collectors are the first link in the production chain of the carnaúba wax powder. Currently, wax production is centered around two manufacturers in the region, but in the past, local people used to produce it. The economic importance of *C. prunifera* is the main factor for the intense extraction and management of this species.

**Table 1:** Quantities and values of the products from the extraction of carnaúba (*Copernicia prunifera*) in two rural communities in the northeastern semiarid region

	Bem Quer community	Cana community
Quantity of straw leaves (in thousands of leaves)		
Extracted per year	150 to 600 thousand	200 to 900 thousand
Extracted per day	20 to 30 thousand	20 to 50 thousand
Quantity of leaves necessary to obtain 1 kg of powder	1000 leaves	1000 leaves
Values (R$/US$)		
Sale of the powder from the open leaves—each kilogram	R$ 2.50 to 3.00 (US$ 0.59 to 0.71)^a^	R$ 5.00 (US$ 1.20)^a^
Sale of the powder from closed leaves (carnaúba “eye”)—each kilogram	R$ 5.50 to 6.00 (US$ 0.76 to 1.44)^a^	R$ 7.00 to 8.00 (US$ 1.68 to 1.70)^a^

^a^Dollar quote for November 2019

## Discussion

### Socioeconomic data, knowledge, and use of *C. prunifera*

The carnaúba leaf (*C. prunifera*) was the most important part of the plant and had the most indications of uses. Although the leaf (straw) has had several indications of uses, the main category was economic, that is, the pure extraction of straw leaves to remove the powder, and later commercializes it. This data corroborates the study by Vieira and Loiola [36] who indicated the economic importance of carnaúba straw by traditional artisans in the northern state of Piauí. The importance of the extraction powder, production wax, and carnaúba leaves was also observed in the study by Sousa et al. [37], also indicating the fruit and stem as important parts of the species. The versatility in relation to the forms of use makes carnaúba a species of great economic and cultural importance in its occurrence region [31]. *C. alba* (Morong ex Morong and Britton), a co-genus of the carnaúba, also has similar characteristics regarding ethnobotanical aspects, such as the use of leaves for construction, crafts, and animal feeding [38–41].

Furthermore, other tree species have also stood out economically for many local populations. For example, Sousa Júnior et al. [42] indicated the production of pequi oil (*Caryocar coriaceum* Wittm) from the fruit as a category with as much importance as food; its commercialization is so relevant because the oil can be stored during the period between harvests. Another species that has great economic relevance is the umbu (*Spondias tuberosa* Arruda), whose fruit commercialization guarantees an income for many rural communities in the semiarid region [43]. In several regions of the world, species of the *Moringa* genus have different uses, from their leaves and fruits, and are economically important due to their applications in nutrition, industries, and medicine [44, 45].

In several ethnobotanical studies, the plant species studied are presented as having various categories of uses, according to the local people who use and manage them. In addition, depending on the species, the target part of extraction, use, and management may be the fruit (as the examples indicated above), leaf, seeds, stem, tubers, etc. [6]. It is important to note that in many communities, people use different types of plants, some of which have a greater potential for treating diseases (medicinal use), others for subsistence or alternative food (food use), in the production of charcoal, construction of houses, or as wood for combustion (wood use). In a way, all of these categories mentioned above have implications in the socioeconomics of local populations.

Therefore, some studies sought to understand the influence of socioeconomic factors on the knowledge and uses of plants, and some made various types of associations, beginning with general plant use [46–48] to categories of specific uses, such as medicinal [49–51] and food [52]. In general, studies on this focus have varied according to the approach of each researcher. Income and education have been addressed in studies on useful plants in general, which suggest that knowledge decreases with the increase in schooling [53, 54] and increased income [48, 55, 56]. Our data, however, did not show a relationship between socioeconomic variables (length of residence, time in extractive activity, and age) with the extraction of carnaúba straw, with the exception of the income obtained from extraction and the number of leaves extracted, which had a significant relationship, indicating an effort to maximize straw collection, including through group organization, according to local extractors. This reinforced the economic importance of carnaúba leaves as one of the factors that drive the use and management of this species locally. Sousa Júnior et al. [12] found a similar situation for *C. coriaceum*, which showed a great economic importance from the nutritional use of its fruit, the pequi. The same was observed for *S. tuberosa* through the study by Lins Neto et al. [57], which demonstrated the commercial importance of the umbu fruit as a factor that influences the management of this species. In the case of carnaúba, what is strongly different is the target of human selection (the leaf) compared to other studies in which the tendency has been for fruits as targets for selection. This can indicate the economic factor as the predominant force that directs the management of native plant species in socio-ecological systems.

In regard to age, according to the results, we did not observe an influence of this variable on the use and management of carnaúba straw. Similarly, Sousa et al. [48] demonstrated that the local importance of species from the region of Floresta do Araripe, in Ceará, was not influenced by socioeconomic factors, with the exception of a smaller number of species which had age as a factor that explained the importance of some culturally salient species. Moreover, according to these authors, younger people were motivated mainly by the commercial potential of some of the native forest resources, giving them greater local importance. However, for other palm species, age was indicated as a factor that influences leaf collection. Virapongse et al. [25] found that extractors of the *Mauritia flexuosa* L.f. leaf were predominantly young, due to the latter being better able to climb the palm trees to remove the leaves. Campos et al. [19] also demonstrated similar results, indicating that age differences may influence the level of involvement in the collection of Ouricuri leaves (*Syagrus coronata* [Mart.] Becc.). In addition to age, gender is also a factor analyzed in ethnobotanical studies; however, the present study observed that only men perform extractive activities of carnaúba in the communities studied, which did not allow for gender analysis.

However, it is important to note that some species have a greater economic appeal, either through their direct commercialization or from products extracted from it. Thus, the socioeconomic aspect may be one of the driving forces based on the economy [58] that directs the use and management of plant populations of human interest. On the other hand, the economic importance of a species can lead to overexploitation of the resource, harming its potential. Destructive practices in the fruit collection of *Mauritia flexuosa* ( Lf), a species of great cultural and economic importance, are an excellent example of this exploitation [59, 60]. Thus, analyzing the socioeconomic factors related to the knowledge, uses, and management of plant species of economic importance, as well as their sustainable management, will be important for future studies.

### Local management and morphological perception of *C. prunifera*

Data on the perception of extractors are in line with the main results found for native plant species where local people exercise some type of management. The perception of morphological and organoleptic characteristics is an important resource that allows people to manage species of interest, favoring those characteristics that vary between plant populations [12, 57], and thus constituting the basic principle of the artificial selection process [6, 61, 62]. Thus, artificial selection is a criterion that can be based on the perception of characteristics preferred by people, consequently reflecting on the management practices that drive the morphological and genetic divergences between natural and managed populations [63, 64]. The color and size of fruits, for example, are target characteristics of human selection, a trend observed in several studies [9, 12, 57, 64, 65], which can consequently favor the incipient domestication process.

Another morphological structure of plants that has been analyzed from this perspective is the leaf [66, 67] indicating that the color and size and arrangement of the *Agave* leaves are an important attribute of the species that help producers of “pulque” (a drink prepared with *Agave* sap in Mexico) in different stages of production. Although the tuberous root is predominantly the target of selection, the leaves of *Manihot esculenta* ssp. *esculenta* (Euphorbiaceae) (Crantz), can be the target [6, 68]. One of the main studies on leaf management of the Arecaecae family was the influence of leaf management on individuals of butiá (*Butia capitata* [Mart.] Becc) in southern Brazil [18]. According to these authors, traditional management seems to be the most interesting both from the point of view of the response of the palm, and also from the productivity of leaves for exploitation. Moreover, according to that study, the artisans of the butiá recognize three distinct types of leaves: new apex leaves, which must remain on the plant, while the rest may be extracted; new leaves for making artisanal products; and discarded leaves, as they are not suitable for handicrafts (either due to their rigidity, stains, or dryness) [18].

These data reinforce the importance of the perception that local extractors have about the resources they manage in different locations. Similar to the study by [18], in this present study on carnaúba (*C. prunifera*), the interviews also indicated that extractors perceive different types of leaves, which are locally called “eye of the carnaúba,” two larger (larger new leaves) and one smaller (new leaf that is left on the plant to assist in its regeneration). Our hypothesis that people would perceive morphological diversity in carnaúba, therefore, has been confirmed. Thus, the local perception, especially of morphological characteristics of interest, may indicate the important role of intentional human action in the selection criteria, use (and preferences), and management of plant species of economic importance, especially by having a more conscious (intentional) selection [12, 69]. A logical consequence of this understanding is that the management of species of economic importance in socio-ecological systems (integration of socioeconomic and biophysical processes and components) [70] is the driving force behind targeting characteristics of human interest [12].

Some studies already carried out on the carnaúba itself indicate the great importance that this species presents, thus justifying the intense management exercised over it in a large part of northeastern Brazil. Vieira et al. [71] indicated that the mensal extraction of immature leaves reduces the production by 50 to 70% of flowers, fruits, leaves, and seeds. Ferreira et al. [29] indicated that the best leaf-cutting management strategy is a single annual cut in December; that way, strategies for the sustainable extraction of *C. prunifera* straw can be carried out. According to Sousa et al. [37], the price of powder extracted from the “eye” of carnaúba was estimated at R$ 7.00 (thereabout US$ 1.68 based on the month of November 2019), while the powder removed from ripe straws cost around R$ 5.00 (thereabout US$ 1.20 based on the month of November 2019). Similar data were observed in our study.

The correlation analysis corroborates the interviewees’ own view, recognizing that, despite the morphological preferences for larger leaves and powder extracted from new leaves (the carnaúba eye), the important thing is to have a large number of leaves collected over the period harvest. That indicates that biological, economic, ecological, and cultural factors contribute jointly to the maintenance local management of plant species. A possible consequence of this is the variation in prices of leaves and powder caused by the increased supply of those products, resulting from intensive collection activities. Thus, extractors may have their income impaired due to the fall in product prices at the beginning of the production chain [72]. Another possible negative impact is the ecological effects on the resource [73], since the intensity of leaf extraction (especially immature) can reduce the size and biomass of reproductive and vegetative structures, according to Vieira et al. [71]. One of the implications of this is the importance of seeking strategies for the sustainable use of biodiversity resources of economic importance, such as the carnaúba leaf, in order to match the conservation of natural resources with income for human populations that use such resources [74, 75].

Therefore, it is important to emphasize that regression analyses further reinforces the economic importance of carnaúba leaves as one of the factors that guides the use and management of this species. Sousa Júnior et al. [12] found a similar situation for *C. coriaceum*, which had a great economic importance from the nutritional use of its fruit, the pequi. For carnaúba, what is strongly different is the target of human selection, which in this case is the leaf, compared to other studies in which the tendency has been for fruits as targets of selection. This can indicate the economic factor as the predominant force that directs the management of native plant species in socio-ecological systems. Nevertheless, it is necessary to develop studies that seek to ascertain this hypothesis, as well as the production chain associated with other socio-cultural factors that may be related to the use and management of carnaúba.

## Conclusions

*Copernicia prunifera* (carnaúba) demonstrated relevant economic and cultural importance for the local populations in the communities of southern Maranhão, northeastern Brazil. The management of the species is associated with its leaves’ strong economic value, due to the wax powder extracted from the carnaúba straw. This seems to be the main factor that favors the local management of carnaúba. Although local extractors perceive leaf variations (target of extraction), management directed to such variations was not observed, as the collection efforts are directed to larger quantities, thus guaranteeing higher income. Thus, the straw collection involved the formation of groups of collectors in one of the communities, in which the extractors were the specialists.

The collection and protection of *C. prunifera* individuals were the main forms of management indicated by the extractors. Despite the indication to collect all the leaves, one of the management strategies indicated by the carnaúba interviewers was to stop collecting leaves from some individuals so that they would have leaves with greater powder production. Local extractors also observed that new leaves (“carnaúba eyes”) have the highest sales value, as they have the highest production of powder. The formation of groups of extractors is another aspect directly related to the amount collected. It is possible that the importance of the economic value of many native species is the predominant factor in their management strategies, as was observed for carnaúba.

## Data Availability

The datasets used and/or analyzed in the current study are available from the corresponding author on reasonable request.
